# Fake news and fallacies: Exploring vaccine hesitancy in South Africa

**DOI:** 10.4102/safp.v63i1.5345

**Published:** 2021-10-26

**Authors:** Avania Bangalee, Varsha Bangalee

**Affiliations:** 1 Department of Medical Virology, Faculty of Health Sciences, Prinshof Campus, University of Pretoria, Pretoria, South Africa; 2 National Health Laboratory Services, Johannesburg, South Africa; 3 Discipline of Pharmaceutical Sciences, Faculty of Health Sciences, University of KwaZulu-Natal, Durban, South Africa

**Keywords:** vaccine hesitancy, vaccines, public health, COVID-19, vaccine acceptance, immunisation, South Africa

## Abstract

Historically, vaccine hesitancy (VH) has been a thorn in the side of public health efforts to contain and eradicate infectious diseases. This phenomenon is magnified in light of the current coronavirus disease 2019 (COVID-19) pandemic. Surveys conducted across South Africa since the outbreak of COVID-19 demonstrate the complexity of factors that contribute towards VH in this population. Amidst the negative press that the COVID-19 vaccine has received, especially across social media, understanding and combatting VH remains important to achieve herd immunity. This article aims to shed light on key factors fuelling COVID-19 VH in South Africa and provides a framework from which to address this problem.

## Background

It has been well over a year since the first case of the coronavirus disease 2019 (COVID-19) was reported in Wuhan, China. Apart from strict government mandated mitigation measures across South Africa (SA), there has been no breakthrough cure. Consequently, the use of unregulated medicines to either treat or prevent COVID-19 has become widespread amidst these heightened concerns. The development of a vaccine was therefore expected to be the answer so many people had been waiting for. The first phase of vaccines in SA was rolled out in February 2021 after safety and efficacy approval from the South African Health Products Regulatory Authority (SAHPRA) was obtained. Since then several strategies were implemented to increase vaccine accessibility. These included the up-skilling of the current health workforce to meet the immediate demand for vaccinators and the provision of vaccines through both the public and private healthcare sectors.^1^ Despite government’s best efforts, vaccine hesitancy (VH) is a growing threat that may prove to be SA’s Achilles heel in the efforts to contain the pandemic.

## What is vaccine hesitancy?

The World Health Organization (WHO) has defined VH as a ‘delay in acceptance or refusal of safe vaccines despite availability of vaccine services’.^2^ Vaccine hesitancy is a worldwide phenomenon and evidence suggests that it has become more acute in recent years.^3,4^ In higher income settings such as the United States (US) and Europe, VH has led to vulnerable pools of unimmunised children resulting in large measles outbreaks.^5,6^ Similarly, across Africa vaccination programmes are challenged by VH, for instance, disinformation and public distrust in vaccination had led to a fivefold increase in polio incidence in Nigeria between 2002 and 2006 and subsequent polio outbreaks across three continents.^7^ The VH phenomenon is magnified in light of the current COVID-19 pandemic.

Vaccine hesitancy in the South African context can be demonstrated since the outbreak of COVID-19 from responses to several online studies, which were conducted to assess vaccine acceptance and hesitancy.^8,9^ Across all studies, vaccine acceptance levels ranged from 82% to 52%. Results from these studies further demonstrated the complexity of factors that contribute to VH in SA. Broadly, drivers of decreased confidence in COVID-19 vaccines include several factors. These include socioeconomic factors such as inequities in access to healthcare, education and accurate information. In addition, disinformation and conspiracy theories through social media and a lack of targeted campaigns and effective public health messaging also contribute to lowered confidence in the vaccine.^10,11^

Results from the University of Johannesburg (UJ)/Human Sciences Research Council (HSRC) COVID-19 Democracy Survey, the most comprehensive study to date on this topic, suggest that approximately a third of the adult South African population is hesitant to receive the COVID-19 vaccine, a proportion that is higher than for most countries globally.^9^ The most cited reason for a lack of acceptance is concern over vaccine safety followed by a low confidence in the effectiveness of the vaccine. In fact, a few reports now suggest an increase in VH following the AstraZeneca safety concerns recently raised in Europe and Africa.^12^

In SA, VH can in part be rooted in its apartheid colonial rule.^13^ Historically, Africans were often the victims of unethical testing in the name of scientific advancement. This viewpoint has recently reared its head because of comments shared by French doctors Jean-Paul Mira and Camille Locht, who suggested that COVID-19 vaccines should be tested in Africa, where ‘there are no masks, no treatments, no resuscitation’.^14^ These provocative comments generated an uproar across the globe for their racist sentiment. Media reports of this kind fuel the distrust that many Africans feel towards the COVID-19 vaccine. Furthermore, with the country forced under lockdown restrictions for the greater part of 2020, the majority relied on online and social media platforms as a source of information. In contrast to traditional news media platforms, content on social media is unadulterated and seldomly scientifically vetted, resulting in an infodemic of misinformation, fake news and conspiracy theories surrounding the vaccine. Some of the common conspiracy theories that have dominated social media include the belief that the vaccine will be used to kill Africans so that Western countries can control the continent’s natural resources or as a ploy by Bill Gates to implant microchips in the COVID-19 vaccine in order to control the world.^15^ This misinformation is intensified when propagated by popular figures, trusted community members and leaders.

Vaccine development is a long, complex process, traditionally taking approximately 10–15 years. Coronavirus disease 2019 has exemplified the use of newer technologies and scientific advancements, to fast-track the research, development and manufacturing processes of new vaccines in a much shorter timeframe; in less than 12 months to be precise. Ironically it is these rapid scientific advancements that have contributed to the lack of confidence in vaccine development and concerns surrounding the safety and efficacy of the vaccine. For example, global use of the novel messenger RNA (mRNA) vaccine has created the fallacy that administration of this vaccine may actually alter one’s DNA, creating a post-apocalyptic world of genetically modified humans.^16^

## Addressing vaccine hesitancy in South Africa

Recognising these barriers to vaccine uptake is crucial in understanding how to combat them. Although approaching VH is a complex process, current authoritative literature seems to suggest that efforts to increase vaccine uptake should be focused on those who are hesitant or distrusting of the vaccine rather than antivaxxers or denialists.^17,18^

To this end we propose addressing VH in SA using three pillars: vaccine literacy campaigns, an effective communication strategy and creating an enabling environment.^19^

### Vaccine literacy campaigns

These targeted strategies provide public education, which can be tailored to specific concerns, for example, COVID-19 vaccine safety information. This may be in the form of multimedia campaigns using radio, television, social media and written platforms and can be adapted into suitable languages depending on the target audience.^20^ Tailored education campaigns have been beneficial when targeted to specific attitudes, beliefs and experiences. At a national level, these campaigns need to be approached in a non-stigmatising way, using factual information, which directly addresses concerns raised around COVID-19 vaccines. Owing to the complexity behind the reasons for VH, it is imperative that more studies are conducted to determine which specific population groups and vaccines need to be targeted, as well as to determine the nuanced reasons for VH in certain populations. The SA Government has a crucial role to play by providing financial support and co-operation for these programmes.

### An effective communication strategy

This needs to provide balanced and transparent information to generate greater public confidence. It should be delivered by trusted contingents such as scientific and clinical authorities. Communication in association with a particular political party or viewpoint should be strongly discouraged. Healthcare workers are a reliable source of vaccine information and can therefore influence vaccine uptake at both an individual and population level. As ‘community champions’ healthcare workers may facilitate community engagement and help to guide household decision makers.^21^ An effective communication strategy to counter hesitancy must be based on sound, evidence-based information within a local context. Moreover, timely communication in addressing concerns arising during the vaccine roll-out will mitigate any false assumptions that may decrease public confidence. To this end, it is essential to identify and involve trusted, credible information sources across SA, when developing and providing vaccine information.

### Creating an enabling environment

This third modality requires critical engagement from the government, civil society, faith leaders and the media. By ensuring an efficient COVID-19 vaccine roll-out strategy, positive public sentiment is maximised. For example, vaccination could be offered in the evenings or on weekends and vaccination sites such as places of worship can offer familiarity in communities that are distrustful of the government. Transportation services provided to communities that are far from vaccination sites may offer a solution to people who may not have considered vaccination as an option. In addition, easily available online material for vaccine recipients and healthcare workers will help to create positive user feedback and strengthen the vaccination programme.

## Conclusion

The South African government has remained resolute that the COVID-19 vaccine will save lives. Deviating from this message could have devastating effects. Sadly, the death toll is expected to rise until vaccine coverage is sufficient to interrupt widespread community transmission of the virus. The propagation of fake news and fallacies across social media concerning the COVID-19 vaccine may gravely reduce confidence in the vaccine. It therefore remains the responsibility of the healthcare and scientific community to determine and address the reasons behind VH both at a community and national level. Solidarity in the message that is relayed to the public regarding the safety and legitimacy of vaccines is now the cornerstone to overcome VH in the greatest pandemic of our time.
